# IceBreaker: Software for high-resolution single-particle cryo-EM with non-uniform ice

**DOI:** 10.1016/j.str.2022.01.005

**Published:** 2022-04-07

**Authors:** Mateusz Olek, Kevin Cowtan, Donovan Webb, Yuriy Chaban, Peijun Zhang

**Affiliations:** 1 Electron Bio-Imaging Centre, Diamond Light Source, Harwell Science and Innovation Campus, Didcot OX11 0DE, UK; 2 Department of Chemistry, University of York, York, UK; 3 Division of Structural Biology, Wellcome Trust Centre for Human Genetics, University of Oxford, Oxford OX3 7BN, UK; 4 Chinese Academy of Medical Sciences Oxford Institute, University of Oxford, Oxford OX3 7BN, UK

**Keywords:** cryo-EM, single particle, vitreous ice, ice gradient, ice thickness, particle picking, preferred orientation, density map

## Abstract

Despite the abundance of available software tools, optimal particle selection is still a vital issue in single-particle cryoelectron microscopy (cryo-EM). Regardless of the method used, most pickers struggle when ice thickness varies on a micrograph. IceBreaker allows users to estimate the relative ice gradient and flatten it by equalizing the local contrast. It allows the differentiation of particles from the background and improves overall particle picking performance. Furthermore, we introduce an additional parameter corresponding to local ice thickness for each particle. Particles with a defined ice thickness can be grouped and filtered based on this parameter during processing. These functionalities are especially valuable for on-the-fly processing to automatically pick as many particles as possible from each micrograph and to select optimal regions for data collection. Finally, estimated ice gradient distributions can be stored separately and used to inspect the quality of prepared samples.

## Highlights

• Develop a software tool for image segmentation based on estimated ice thickness
• Present a method to detect and annotate ice contamination in the dataset
• Show a procedure to equalize contrast on the micrographs with the non-uniform ice
• Demonstrate a workflow to identify optimal ice for data collection/particle selection

## Graphical abstract

**Figure:**
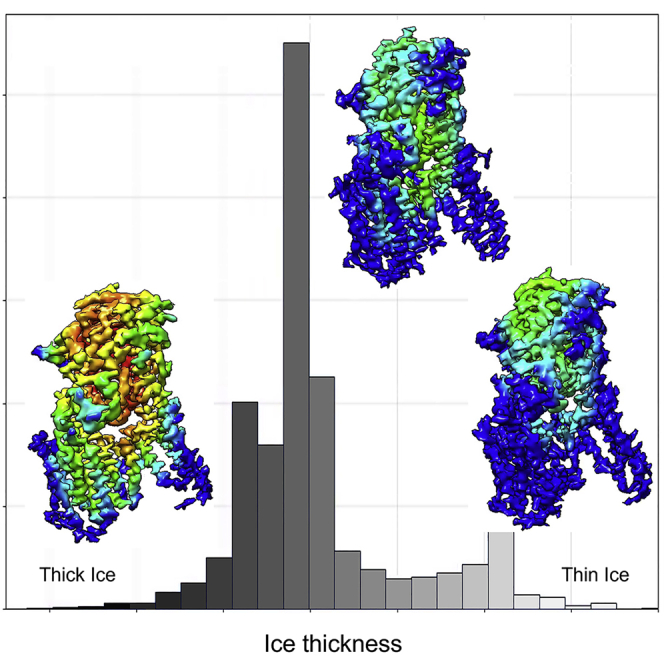

## Teaser

Olek et al. present a software tool, IceBreaker, for handling non-uniform ice thickness in cryo-EM micrographs. Ice thickness is believed to be a crucial factor that affects the quality of cryo-EM reconstructions. IceBreaker provides empirical estimation of the ice distribution and introduces an ice thickness parameter to the cryo-EM processing pipeline.

## Introduction

Advancements in cryoelectron microscopy (cryo-EM) instrumentation, detector development, and data processing algorithms have allowed reconstructions to be obtained at atomic resolution (Nakane et al., 2020). The final quality of the cryo-EM reconstruction depends on several factors at different stages from the sample preparation and data collection to the data processing. One of the crucial features is the thickness and variance of the vitreous ice across the grid. The ice parameters in principle can be optimized at the sample preparation stage by the adjustments of plasma exposure time, blot force, and time (Passmore and Russo, 2016). Despite recent advancements in instrumentation, the vitrification process is still highly variable and not reproducible (Dandey et al., 2020; Drulyte et al., 2018; Rubinstein et al., 2019; Tan and Rubinstein, 2020). The overall quality of the prepared cryo-grids needs to be assessed before the data collection. Currently, user tools in data collection software such as EPU can be helpful in the automated selection of the best areas of the grid and excluding damaged areas. More advanced routines to estimate the ice thickness using energy filter, the aperture limited scattering method (Rice et al., 2018), diffraction patterns (Ahn et al., 2020), or classification routines based on machine learning algorithms for the images at low magnification (Yokoyama et al., 2020) allow targeting only the grid areas with desired ice thickness. This can lead to improvements in the final resolution and reduce the data collection time, but most of the methods need to be optimized for each project and microscope (Rheinberger et al., 2021).

The ideal setup for single-particle analysis would have the particles distributed in a thin, vitreous ice layer. The surface of the ice in the data collection areas should be flat and normal to the electron beam. Particles should occupy most of the grid holes, be oriented randomly, and not overlap with each other (Noble et al., 2018). Areas with too thin ice can be devoid of proteins or the proteins can be damaged or denatured on the air-water interface (D’Imprima et al., 2019). Thicker ice results in low SNR, errors in defocus determination, and limits the final resolution. Even though it is recommended to make the grids with thinnest-possible ice that can still support the specimen, in many cases the particles will be pushed to thicker ice areas (Wu et al., 2016), or, in other cases, the particles will have preferred orientation(s) (Cianfrocco and Kellogg, 2020; Glaeser and Han, 2017). Generally, the collected dataset will include images of variable ice thickness that affects signal-to-noise ratio (Baxter et al., 2009). Recently, image processing techniques or artificial intelligence-(AI)-based denoising software tools have been developed to improve the interpretability of the micrographs (Bepler et al., 2020). The denoised micrographs allow for picking additional particles that were otherwise not distinguishable from the noise (Wagner and Raunser, 2020). The problem of preferred orientation and missing angular projections of the specimen can limit the final resolution and affect the performance of the map reconstruction algorithms even with the large number of picked particles (Rosenthal and Henderson, 2003; Sorzano et al., 2021).

One main shortcoming common to most of the state-of-the-art automated tools is the fact that they do not take into consideration the fact that particles distributed in different ice thickness regions may have different quality and features. After the processing, most of that information, which could lead to the improvement of the final resolution, cannot be recovered. Currently there is no software tool that allows the user to easily connect the ice thickness parameter with the quality and state of the particles in different areas of the prepared sample.

In this work, we present a software tool, IceBreaker, for the ice thickness estimation and digital ice gradient removal on the cryo-EM micrographs. The software allows the segmentation of the micrographs and grouping areas with similar ice features. It can be used for local image processing as filtering or contrast enhancement, as well as annotating and removal of the ice contamination and/or carbon film fringes. Importantly, it introduces the empirical ice thickness parameter that can be associated with each particle based on the picked coordinates. The described tool can be used as a stand-alone image processing software or as an external job in the integrated Relion workflow (Zivanov et al., 2018).

## Results

### The IceBreaker workflow

**Figure 1:**
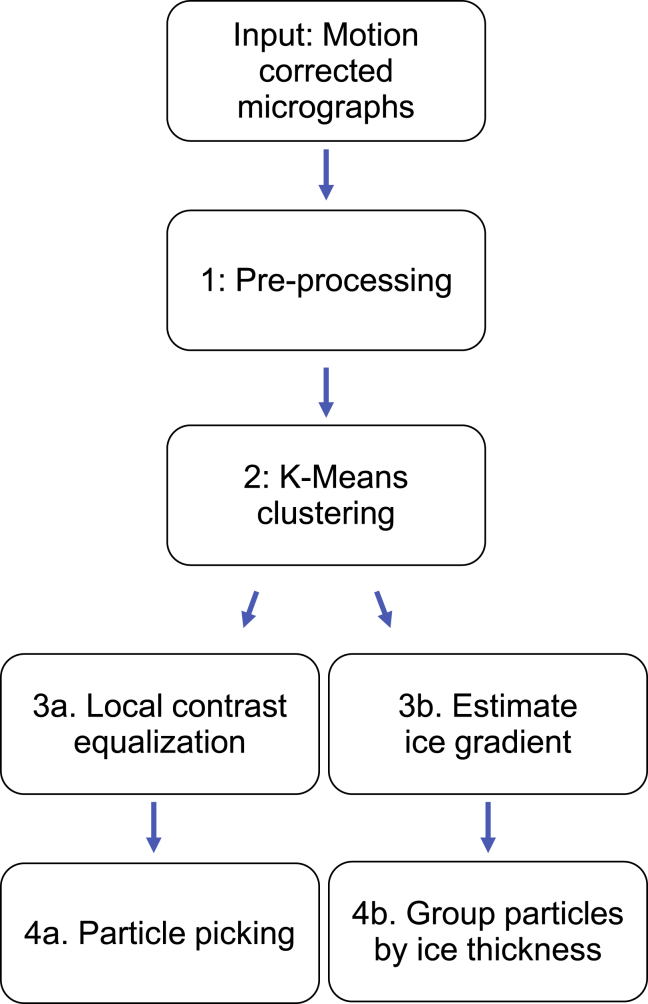
The IceBreaker workflow The required input is a set of motion-corrected micrographs. The pre-processing stage includes low-pass filtering and further feature flattening done by local averaging. The output image is used for the K-means clustering to obtain segmented micrographs. From the segmented micrographs, the user can create local masks for local contrast improvement, which can lead to improved particle picking, or empirically estimate ice gradient and use this information as an additional parameter for the processing.

The IceBreaker software allows segmentation of the cryo-EM micrographs based on the distribution of the pixel intensities recorded by the detector. The term “estimated ice thickness value” is introduced to describe and group the areas of the micrograph with similar pixel intensities. This information can be exploited during the later stages of the cryo-EM processing pipeline; e.g., particle picking, 2D classification, or 3D refinement. An overview of the workflow is presented in Figure 1 with examples of the resulting images. Each of the steps is described below.

Input data: the required input is a set of motion-corrected cryo-EM micrographs. The IceBreaker can be run as an external job of the Relion project or as a stand-alone tool from the command line. It can be used as a part of the data collection pipeline or performed on historical data. Various tools for motion correction (Grant and Grigorieff, 2015; Li et al., 2013; Zheng et al., 2017; Zivanov et al., 2019) can be installed separately. They do not affect IceBreaker results, as long as the whole dataset is processed with the same setup. The pixel intensity values from the input images are used to estimate the distribution of the ice thickness in a given dataset.

Step 1. Pre-processing: filtering and feature flattening: the 20 Å low-pass filter is applied to each micrograph to remove the high-frequency noise and reveal features such as particles, ice contamination, foil hole edges, and the ice gradient. Then, the micrograph is divided into a pre-defined number of patches: 40 in x and 40 in y direction, which is independent of the size of the micrograph. Within each patch, an average value of pixel intensities is calculated. This way local features are reduced to 1/1,600 of the micrograph area on top of the initial 20 Å filter. In our test cases, this was sufficient to reveal trends and low-frequency changes in the background, which represent the changes in the ice thickness. Additionally, the super-pixels represented by each patch can be used to reduce the size of the micrographs and improve the computation speed. Micrographs processed this way are used as input to the next stage of the processing.

Step 2. K-means clustering: the K-means clustering algorithm is used to group together the areas of the rescaled, feature-flattened micrograph with similar values. By default, each micrograph is divided into 16 segments. Then, the segmented image is upscaled to match the original size of the micrograph. This results in a micrograph with 16 discrete regions with unique values of the intensities of the pixels. Each group populates the pixels that originally represented similar background features in a given neighborhood. The segmented micrographs are saved and can be used for further processing in two ways. First, for masking and local processing of the original micrographs, and second, as a reference to identify the micrograph quality in the neighborhood of the coordinates selected during the particle picking.

Step 3. Local processing for contrast improvement or ice gradient estimation: the groups defined in the previous step allow the local processing of the original dataset. Each segment represents an area with similar background features and can be used as a local mask that can be applied to the original, motion-corrected micrographs. Within each mask, the image processing operations such as contrast improvement can be performed. The application of the contrast equalization in different areas of the micrograph separately results in the final image with a similar ratio between the particles and the background features. This also alleviates the problem of oversaturation of parts of the image when it is equalized as a whole. The resulting image has a similar ratio between the particles and the background, which can be beneficial for the particle picking tools based on the template matching algorithms.

Another use of the presented approach allows estimating the average ice thickness in segmented micrographs. The defined local masks can be applied to the motion-corrected images. Within each mask, an average value of the pixel intensities can be calculated to estimate the ice thickness in the selected region. This way, a set of segmented micrographs with the estimated ice distribution is created and can be used to associate the picked particle coordinates with the background intensity in the area where they come from. The empirical ice thickness parameter describes whether the particle was picked from the area with high signal-to-noise ratio (which would correspond to the thin ice conditions) or low for the particles embedded in thicker ice. It also allows filtering and selecting subsets of particles of similar quality.

The performance of the IceBreaker was tested using several datasets available from the Electron Microscopy Public Image Archive (EMPIAR) database. The presented results are focused on the main features of the software: (1) local contrast enhancement to improve the particle picking; (2) evaluation of the micrographs’ quality and identification of the ice contaminations and foil hole edges; and (3) the cryo-EM data processing with the newly introduced empirical ice thickness parameter.

### Local contrast enhancement

**Figure 2:**
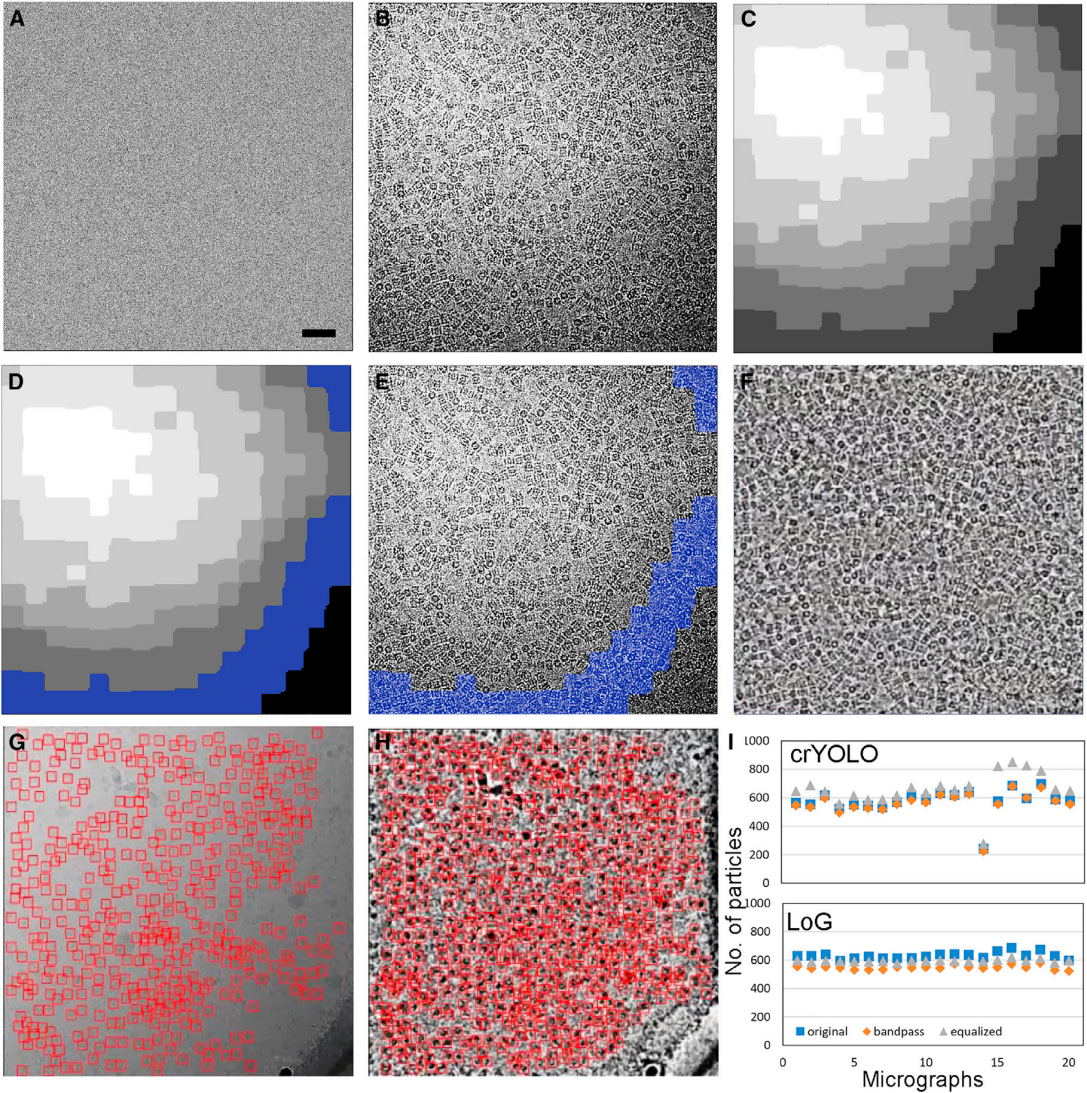
IceBreaker contrast enhancement (A) A raw micrograph of T20S (EMPIAR-10025) used as an input. (B) A 20 Å low-pass filtered micrograph, revealing non-uniform distribution of ice. (C) A segmented micrograph, where each segment can be used as a local mask. (D and E) Local mask (blue) applied to a corresponding example segment of the micrograph. (F) The micrograph after contrast equalization. (G and H) Automated particle picking using crYOLO on the original micrograph (G) and after local contrast equalization (H). (I) Number of particles picked by crYOLO (top) and LoG (bottom) from original (blue), 20–500 Å band-pass-filtered (orange), and local contrast-equalized (gray) images randomly selected from the dataset (10%). Scale bar, 50 nm.

One of the main challenges when processing cryo-EM micrographs with non-uniform ice distribution is the fact that the contrast levels between the particles and the background features vary in different parts of the image. This can affect the performance of the automated particle pickers, especially those using a single value threshold to detect false-positives. In order to normalize the local contrast between the particles and the background across the whole micrograph, IceBreaker segments low-pass-filtered micrographs into areas of similar overall intensity. The procedure of local contrast enhancement is presented in Figure 2. The input motion-corrected micrograph (Figure 2A) is pre-processed using a low-pass filter to identify the changes in background intensities corresponding to the ice distribution (Figure 2B). The K-means clustering is applied to the low-pass-filtered micrograph to obtain a segmented image (Figure 2C) where pixels with similar intensities are grouped together. Each of the segments created this way can be used as a local mask for image processing. An example of such a mask is highlighted blue in Figure 2D. It can be applied to the low-pass-filtered micrograph to directly access pixel coordinates as shown in Figure 2E. Within each mask, the histogram equalization is performed. This procedure is repeated for each segment of the micrograph. The resulting image in Figure 2F is flattened with the ice gradient removed. Contrast between particles and the background features is improved both in the areas that were originally dark and bright. Images curated this way can be used as a direct input for automated particle picking. As shown in Figures 2G and 2H, particle picking with crYOLO is much improved after image flattening and contrast enhancement (Figure 2H) compared with the original micrograph (Figure 2G). The particles initially skipped due to poor contrast are now included, especially those in the darker area, yielding a greater number of picked particles. While increasing the number of picked particles is valuable when the dataset is small, views with weak contrast are missing, or when performing 2D classification, users should keep in mind that the quality of the particles from thicker ice regions might be poorer and should be evaluated when aiming for the best possible resolution. IceBreaker introduces means for such evaluations, which are described below. Figure 2I shows a comparison of the number of particles picked with Relion3.1. Laplacian of Gaussian (LoG) autopicker from original micrographs, micrographs after band-pass filtration (with the setup of 20–500 Å), and micrographs after contrast equalization with the IceBreaker. The IceBreaker produces micrographs with consistent intensity distribution, which allows the pickers to perform more reliably. By contrast, the band-pass filter produces a correction that often varies over the area of the micrograph and does not equalize the contrast between particles in thin and thick areas. The improved picking from band-pass-filtered images is still affected by the changes between the micrographs, such as defocus value, as the filter parameters are set globally for the whole dataset. The IceBreaker allows us to improve the contrast for each micrograph individually and achieve better results.

### Micrograph quality evaluation and ice contamination detection

**Figure 3:**
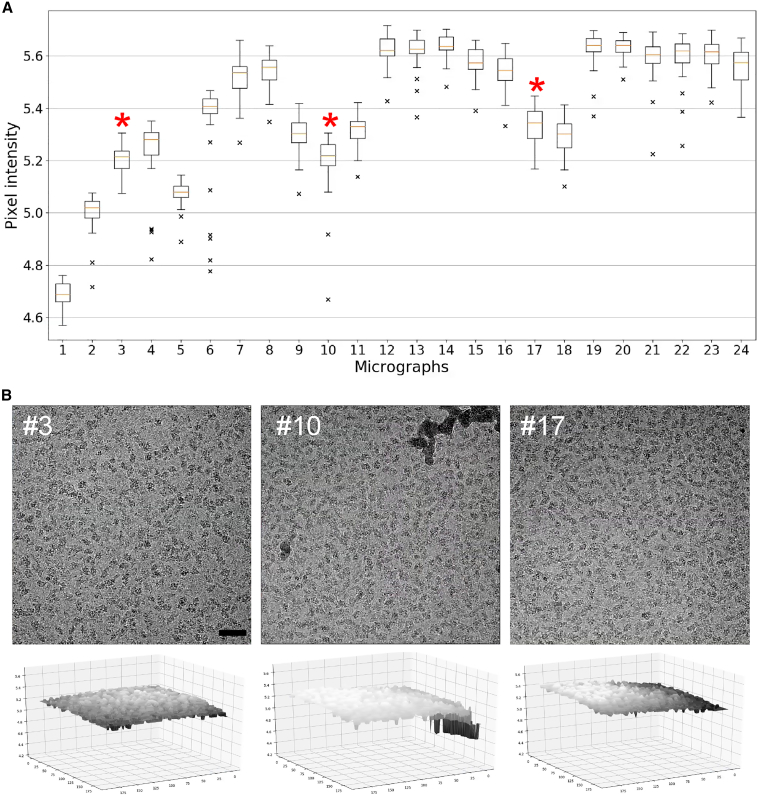
Assess the distribution of the ice by IceBreaker (A) Box plots for a subset of 24 micrographs of β-Gal from EMPIAR-10204, showing pixel intensities distribution in the micrographs after segmentation. (B) Images and corresponding 3D ice distribution profiles of selected micrographs. Asterisks (^∗^): micrograph no. 3 with no ice contamination and uniform ice distribution, micrograph no. 10 with the ice contamination indicated by the outliers on the box-plot, micrograph no. 17 with the non-uniform ice gradient represented by the skewed distribution. Scale bar, 50 nm. The size of each of the boxes in the box plots (equivalent of error bar) corresponds to the values of the first and the third quartile; orange bar represents median value of the given micrograph. The whiskers indicate datapoints that fall into the 1.5 interquartile range (IQR) and the outliers (marked with black X) represent datapoints that significantly differ from the dataset.

The segmented micrographs can be used to evaluate the overall quality of the collected dataset, in addition to CTF estimation. Figure 3A shows the distribution of the pixel intensities, which represents the background for a subset of 20 micrographs from the beta-galactosidase dataset EMPIAR-10204 (Kato et al., 2018). This analysis revealed several features of the data, which are discussed on selected examples of the micrographs and their 3D profiles presented in Figure 3B: (1) micrographs with darker backgrounds, associated with the thicker ice in these areas of the grid, can be easily separated from the ones with a lighter background and thinner ice; (2) a symmetrical box plot indicates a uniform background as in micrograph no.3, while a skewed box plot in micrograph no. 17 or 3D presentation suggests an ice gradient; (3) the outliers in box plot representing micrograph no.10 and the corresponding 3D representation indicate there are ice contaminations. Such analysis provides information that can improve further processing. Micrographs with lower quality can be excluded. The outlier analysis can be helpful to set thresholds for the particle pickers to avoid ice contaminations or remove them from the already-picked set of coordinates. Figure S1A shows a segmented micrograph with the ice contamination in the field of view. The contaminations can be easily identified by checking the pixel intensities distribution (Figure S1B). The coordinates picked with the LoG include areas associated with the contamination, which can be easily removed based on the pixel intensities distribution thresholding (Figures S1C and S1D). Associating the particles' coordinates with local background values can also help to exclude false-positive particle positions with automated pickers based on template matching or machine learning.

### Processing based on the ice thickness parameter

**Figure 4:**
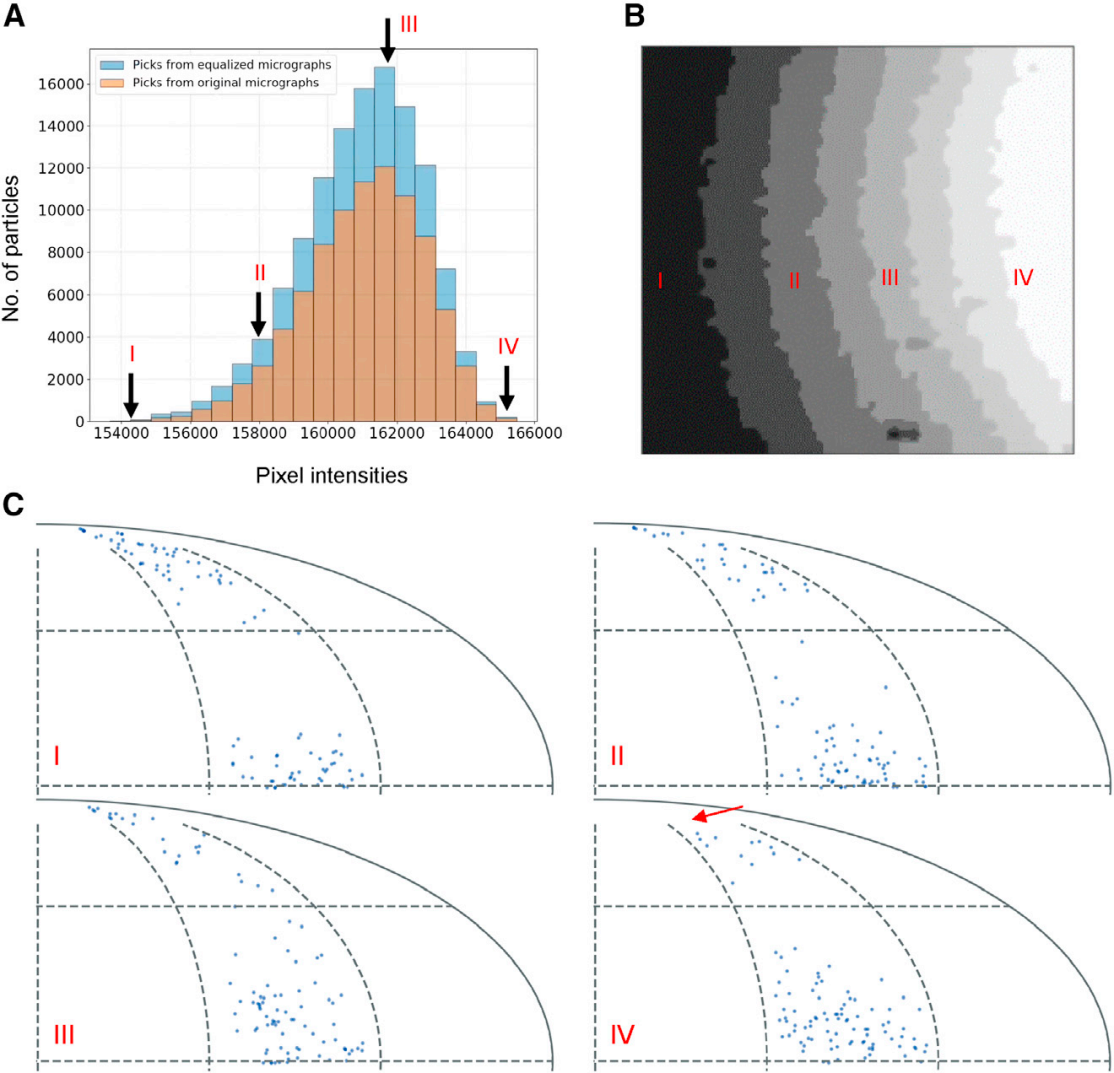
Distribution of T20S particles (EMPIAR-10025) in different ice thickness (A) Distribution of the number particles picked with crYOLO from original micrographs (gold) and from contrast-equalized micrographs (cyan). (B) An example of segmented micrograph with strong ice gradient, from the thick (I) to the thin (IV) ice area. (C) Angular distribution of particles in selected ice thickness areas (I–IV). For each region, 100 particles were selected randomly to match the lowest populated group, I. The red arrow shows that the top views of the particles are not supported in the thinnest ice group, IV, and particles orientation are shifted toward equatorial area.

The information about the distribution of the background pixel intensities can be associated with the coordinates of the particles picked using any available picking tool. With the IceBreaker, we introduce a new empirical particle parameter representing the estimated ice thickness based on the background features of the area where the particle is located. Users can check the overall distribution of the particles and their orientations with respect to their background quality. Figure 4 presents such analysis using the T20S proteasome dataset EMPIAR-10025 (Campbell et al., 2015). The histogram in Figure 4A shows the number of particles associated with different ice thickness values. These values are calculated from the segmented micrographs as an average value of pixel intensities in each segment (Figure 4B). The histogram shows that the majority of the particles were picked from the intermediate ice thickness values. There is an apparent skewness in the particle distribution due to the absence of particles in very thin ice, which is possibly too thin to embed T20Ss proteasome particles. The set of over 120,000 automatically picked particles, after 3D refinement in Relion with the D7 symmetry, were split into 20 groups based on the ice thickness parameter. This allows us to assess how the particles behave in different ice thickness conditions, as shown in the particle angular distribution (Naydenova and Russo, 2017) plots (Figure 4C). For presentation clarity and to match the lowest populated group I, each plot is done for a randomly selected subset of 100 particles. In group I, which represents the thicker ice area, the number of picked particles is low, but both top views and side views of the T20S proteasome are present. As the ice gets too thin to support the top view (group IV), the angular plot shows a shift from the pole (top view) toward the equatorial area (side view). The selection of the particles from the regions can lead to under-representation of specific views, or preferred orientation, even if the signal-to-noise ratio is better. The most populated groups in the intermediate ice thickness show good support for most of the angular views required for an isotropic reconstruction (groups II–III), still the quality of the particles and signal-to-noise ratio may differ between the groups.

**Figure 5:**
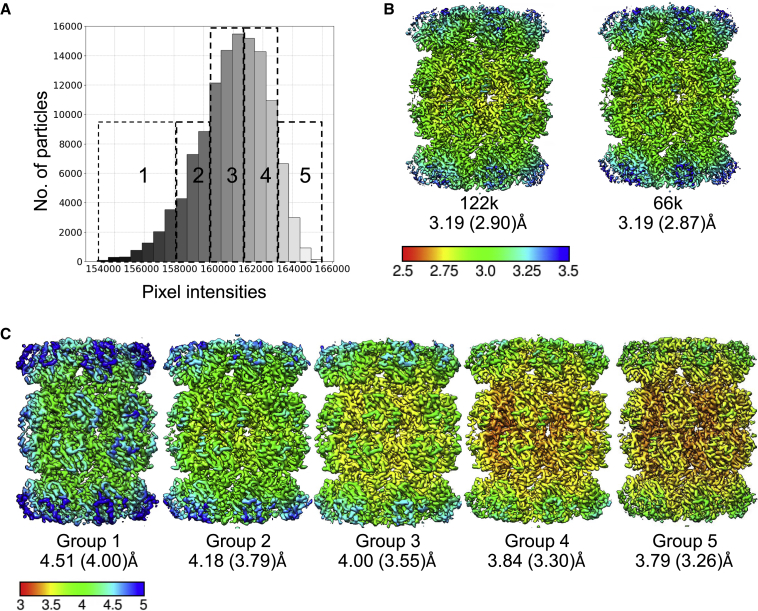
3D reconstruction of T20S particles based on ice thickness (A) Particle subsets selected from the T20s dataset (EMPIAR-10025) according to ice thickness parameter. Group 1 corresponds to the thick ice, group 5 the thin ice. (B) Cryo-EM maps reconstructed from all 121,913 particles and 66,000 particles from thinnest ice groups, 4 and 5. (C) Cryo-EM maps reconstructed from a set of 7,000 particles picked randomly from each ice group 1–5, D7 symmetry applied. Maps are colored according to the local resolution. For each map, reported resolutions after 3D refinement and post-processing are indicated. Temperature scale bar values are in Angstroms.

To further gauge the effect of ice thickness on 3D reconstruction, we regrouped the full dataset of picked particles into five groups based on the ice thickness parameter, as shown in Figure 5A. Figures 5B and 5C show the post-processed maps rendered in UCSF Chimera (Pettersen et al., 2004). Maps are colored by the local resolution calculated with LocRes (Kucukelbir et al., 2014) and labeled with the final resolution for each reported after Refine3D and post-processing jobs. Figure 5B shows a comparison of the densities obtained using all 121,000 particles and 66,000 particles from thinnest-ice groups (4 and 5). Particles from optimal ice conditions allowed to obtain similar resolution, 3.19 Å after refinement and 2.87 Å after post-processing, as the larger number of particles (3.19 Å and 2.90 Å respectively). From each ice thickness group, a random subset of 7,000 particles was selected for an additional round of 3D refinement with D7 symmetry followed by the post-processing with Relion. The setup parameters for each subset were the same, as well as the mask used for post-processing. There is a clear trend that the resolution improves as the ice thickness reduces, from 4.5 Å to 3.8 Å after refinement and 4.0 Å to 3.26 Å after post-processing. This shows that associating the particles with the local ice thickness can help to identify the optimal ice thickness areas to obtain the best possible resolution for a given specimen. This also allows us to test whether preferred orientation may have been caused by recording data from areas of sub-optimal ice thickness. Finally, if the size of the data allows, resolution improvement can be achieved by selecting particles from particular ice groups.

**Figure 6:**
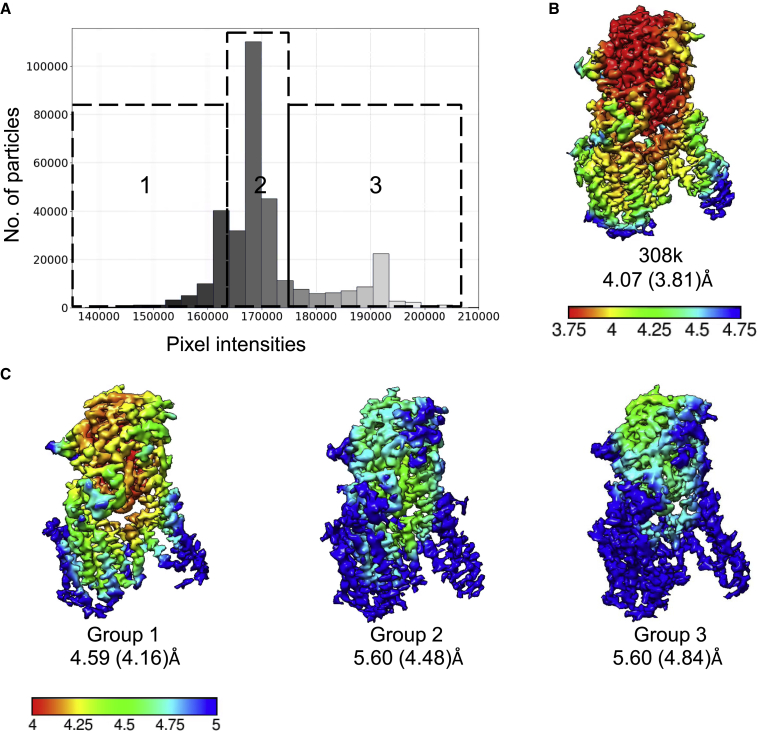
3D reconstruction of gamma-secretase particles based on ice thickness (A) Particle subsets selected from the gamma-secretase dataset (EMPIAR-10194) according to ice thickness parameter. Group 1 corresponds to the thick ice, group 3 the thin ice. (B) Cryo-EM map reconstructed from all selected particles. (C) Cryo-EM maps reconstructed from a set of 60,000 particles picked randomly from each group 1–3, C1 symmetry applied. Maps are colored according to the local resolution. For each map, reported resolutions after 3D refinement and post-processing are indicated. Temperature scale bar values are in Angstroms.

The T20S proteasome has a D7 symmetry and may not be affected by the lower number of edge-on views in thin ice. We therefore selected another low-symmetry particle dataset, gamma-secretase (EMPIAR-10194), for the ice thickness-based refinement (Bai et al., 2015). The distribution of particles in the estimated ice thickness groups was analyzed (Figure 6A). Combining all particles from various ice thickness resulted in a density map at 4.07 Å resolution after refinement and at 3.81 Å post-processing. The particles were later divided into three groups based on the estimated ice thickness value. From each group, a subset of 60,000 particles was randomly selected and refined with C1 symmetry. In this case, a trend of resolution improving with thicker ice allowed to improve resolution from 5.60 Å to 4.59 Å after refinement and from 4.84 Å to 4.16 Å in thick ice after post-processing. This result in conjunction with the previous example shows that particles from different estimated ice regions substantially influence the quality of the cryo-EM map.

## Discussion

The non-uniform ice distribution on the cryo-EM micrographs affects the data processing and the quality of the final map. The thickness of ice in which the particles are embedded affects the local signal-to-noise ratio, particle quality, and behavior. The presented software, IceBreaker, aims to overcome the issues caused by the varying ice gradient. The tailored contrast enhancement can improve the micrographs’ interpretability and the performance of automated particle picking tools. At the same time, it allows the application of information about the ice distribution in the original micrograph for later stages of processing. To our knowledge, currently no other software offers this level of insight into the ice gradient in the micrograph.

With the analysis of pixel intensity distribution in the segmented micrographs, users can get an insight into overall quality of the collected data. This helps the user to easily identify the micrographs with non-uniform ice distribution, ice contamination, and foil hole edges in the field of view. Based on the outlier analysis, a threshold can be applied to exclude areas of poor quality from further processing.

Our software allows determining empirically and associating the ice thickness parameter with each particle. It allows us to select optimal particles and achieve the best possible resolution for collected cryo-EM datasets. It provides users with additional information about the dataset and the possibility to determine angular distribution of particles in different ice gradient regions. Users can filter and group the particles based on the estimated optical density of the micrographs, normally associated with amorphous ice thickness, ice contamination, or foil hole fringes. Presented results using the EMPIAR-10025 dataset as an example show improvement in the final resolution of the map with the particles picked from thinner ice. Because the T20S proteasome has high symmetry, the effect of missing orientations in thinner ice areas was less prominent. The fact that the non-symmetrical gamma-secretase dataset (EMPIAR-10194) has improved resolution of the map from thicker regions shows that the local ice conditions can affect the quality of the final map, and the thinnest ice sometimes has to be avoided. In this case, better results were obtained from thicker ice. This type of analysis can be done during the initial, small-scale data collection to determine the optimal setup for a given dataset and to target the best ice conditions, whether for the optimal angular orientation coverage or for a better signal-to-noise ratio.

The IceBreaker can be run as an external job in an existing Relion3.1 project, as it has been integrated into Relion seamlessly, or run as a stand-alone software. Further integration with data collection pipelines, such as IspyB (Delagenière et al., 2011), can extend the use of IceBreaker for selection of the best regions for data acquisition on the fly, based on specimen properties. The software is being incorporated as a part of the data processing pipeline (Fernandez-Leiro and Scheres, 2017) and the CCP-EM software suite (Burnley et al., 2017). We demonstrate the utility of IceBreaker with a few examples shown here, and the method can be applied to any cryo-EM single-particle dataset, either already collected or being collected.

## STAR★Methods

### Key resources table

**Table:** 

REAGENT or RESOURCE	SOURCE	IDENTIFIER
**Deposited data**		

Beta-galactosidase	(Kato et al., 2018)	EMPIAR-10204
*Thermoplasma acidophilum* 20S proteasome	(Campbell et al., 2015)	EMPIAR-10025
Human gamma-secretase	(Bai et al., 2015)	EMPIAR-10194
		PDB: 5a63
The cryoEM density map of T20s proteasome with various ice thickness, subset 1 (EMPIAR-10025 reprocessing)	This paper	EMD-13309
The cryoEM density map of T20s proteasome with various ice thickness, subset 2 (EMPIAR-10025 reprocessing)	This paper	EMD-13310
The cryoEM density map of T20s proteasome with various ice thickness, subset 3 (EMPIAR-10025 reprocessing)	This paper	EMD-13311
The cryoEM density map of T20s proteasome with various ice thickness, subset 4 (EMPIAR-10025 reprocessing)	This paper	EMD-13312
The cryoEM density map of T20s proteasome with various ice thickness, subset 5 (EMPIAR-10025 reprocessing)	This paper	EMD-13313
The cryoEM density map of T20s proteasome with various ice thickness, subset 4 and 5 combined (EMPIAR-10025 reprocessing)	This paper	EMD-13902
The cryoEM density map of T20s proteasome with various ice thickness, full dataset (EMPIAR-10025 reprocessing)	This paper	EMD-13901
The cryoEM density map of human gamma-secretase complex with various ice thickness, subset 1 (EMPIAR-10194 reprocessing)	This paper	EMD-13903
The cryoEM density map of human gamma-secretase complex with various ice thickness, subset 2 (EMPIAR-10194 reprocessing)	This paper	EMD-13904
The cryoEM density map of human gamma-secretase complex with various ice thickness, subset 3 (EMPIAR-10194 reprocessing)	This paper	EMD-13905
The cryoEM density map of human gamma-secretase complex with various ice thickness, full dataset (EMPIAR-10194 reprocessing)	This paper	EMD-13907

**Software and algorithms**		

Relion3.1	(Zivanov et al., 2018)	https://github.com/3dem/relion
MOTIONCORR2	(Zheng et al., 2017)	https://emcore.ucsf.edu/ucsf-software
CTFFIND-4.1	(Rohou and Grigorieff, 2015)	https://grigoriefflab.umassmed.edu/ctf_estimation_ctffind_ctftilt
crYOLO	(Wagner et al., 2019)	https://pypi.org/project/cryolo/
LocRes	(Kucukelbir et al., 2014)	http://resmap.sourceforge.net
Mrcfile	(Burnley et al., 2017)	https://github.com/ccpem/mrcfile
NumPy	(Harris et al., 2020)	https://numpy.org
OpenCV	(Bradski, 2000)	https://opencv.org
Gemmi	GEMMI - library for structural biology — Gemmi 0.5.2 documentation	https://github.com/project-gemmi/gemmi
Chimera	(Pettersen et al., 2004)	https://www.cgl.ucsf.edu/chimera/
IceBreaker	This paper	https://github.com/DiamondLightSource/python-icebreaker
		https://pypi.org/project/icebreaker-em/
		Zenodo deposition https://doi.org/10.5281/zenodo.5743790

### Resource availability

#### Lead contact

Further information and requests for resources and reagents should be directed to and will be fulfilled by the lead contact, Prof. Peijun Zhang (peijun.zhang@strubi.ox.ac.uk)

#### Materials availability

This study did not generate new unique reagents.

### Experimental model and subject details

All data are generated from the datasets provided in the Key resources table.

### Methods details

#### IceBreaker scripts

The IceBreaker can be run from the command line or as an external job in Relion project. The software includes two main scripts. The ib_job.py can be used for image processing. It requires motion-corrected micrographs as an input. It can be run in two modes: ‘flatten’ to improve the contrast or ‘group’ to estimate the ice thickness in different areas of the micrographs. The number of threads for parallel processing can also be defined with input parameter but is limited by the number of available CPU threads. Example command which can be used with Relion is:ib_job –o Output/Directory/ --in_mics PathToMotionCorrMicrographs.star –mode flatten –j 10: the micrographs listed in the star file will be processed to improve the contrasts. 10 threads will be used to process 10 micrographs at the same time and speed up the processing. The output micrographs will have the same name as input files with suffix ‘_flattened.mrc’.

ib_job –o Output/Directory/ --in_mics PathToMotionCorrMicrographs.star –mode group –j 10: the micrographs listed in the star file will be segmented according to the background pixels intensities. Again, 10 threads will be used to process 10 micrographs at the same time and speed up the processing. The output micrographs will have the same name as input files with suffix ‘_grouped.mrc’.

The second script **ib_group.py** is used to process the star file with particle coordinates and associate them with estimated background quality. As input, it requires a star file with particle coordinates and a set of ‘grouped’ micrographs created in the previous step with ‘ib_job.py’ in group mode. Example command to run ‘ib_group.py’ is: ib_group –o **OutputFile.star** –in_mics micrographs_grouped.star –in_parts particles.star

The output .star file has an additional column with the ‘ice-thickness’ parameter value for each particle. As for now, this new parameter is labelled as ‘_rlnHelicalTubeID’. The star file can be used in Relion to select subsets of the particles in the processing pipeline.

#### Image processing and analysis

The IceBreaker is written in Python 3. The micrographs are processed with the mrcfile package (Burnley et al., 2017). The STAR files are handled with GEMMI. The tool requires NumPy (Harris et al., 2020) and OpenCV (Bradski, 2000) packages for data processing.

The image segmentation is done with the K-Means algorithm (Lloyd, 1982). It is a commonly used clustering algorithm which can give insight into the structure of the data, in this case the micrographs. The n observations are split into k number of sets S, where k≤n. The objective is to group observations in sets in a way to minimize the sum of squared distances (variance) between the observations and the centre of the cluster to which they are assigned, according to the (Equation 1):

(Equation 1) $$\underset{S}{\mathrm{argmin}}{\sum_{i=1}^{k}\sum_{x\in S_{i}}\left\|x-\mu_{i}\right\|}^{2}=\underset{S}{\mathrm{argmin}}\sum_{i=1}^{k}\left|S_{i}\right|VarS_{i}$$

where x denotes observation, S_i_ is a set of observations and μ_i_ represents the mean of points in set S_i_.

The contrast improvement performed in each defined local mask is based on the histogram equalization algorithm. It adjusts the contrast of the input image to evenly utilize the full range of intensities. To do so, the cumulative distribution function (cdf) calculated for the histogram normalized between 0 and 1 has to be linearised to produce a new image with a flat histogram. The (Equation 2) describes the linearised cdf:

(Equation 2) $$cdf_{y}\left(i\right)=\left(i+1\right)Kfor0\leq i\leq L$$

where y is the corrected image, I is the pixel intensity level, K is a constant value and L is total number of intensity levels. The cumulative distribution function is increasing and continuous thus according to the definition of the inverse distribution function, if F^-1^(p), p∈(0,1), there is a real number x that F(x) = p, therefore F^-1^(F(X)) = X (Gilchrist, 2000). The transform which is applied to the original image to obtain corrected image is described with (Equation 3):

(Equation 3) $$y=T\left(k\right)=cdf_{x}\left(k\right)$$

where y is the corrected image, x is the initial image and k is the pixel intensity level in the range [0, L-1].

To evaluate the quality of the micrographs the box plots are used. They provide information about the data distribution based on the five-number summary (Tukey, 1977). It includes the minimum, the maximum, the median and the first and the third quartile. The first quartile (Q1) represents the 25^th^ percentile, which means that 25% of recorded observations have lower value. The third quartile (Q_3_) represents the 75^th^ percentile. The size of the box is determined by the interquartile range (IQR) which is a distance between Q_1_ and Q_3_, IQR=Q_3_-Q_1_. The outliers are detected as observations outside the range:

(Equation 4) $$\left[Q_{1}-1.5IQR,Q_{3}+1.5IQR\right]$$

#### T20S data processing

The deposited dataset was averaged, therefore no further motion correction was performed. The dataset was processed with Relion3.1 pipeline. The parameters of contrast transfer function were estimated with CTFFIND-4.1 (Rohou and Grigorieff, 2015). The motion corrected micrographs had contrast equalized with the IceBreaker for particle picking. The total number of particles picked with crYOLO was 163,630. After manual selection of the best 2D classes from reference-free classification 121,913 particles were used for 3D classification. The best 3D class was used as a reference for 3D refinement with D7 symmetry which resulted in 3.19 Å resolution based on the gold standard FSC = 0.143 criterion. The post-processing with the soft mask created from low-pass filtered initial 3D class and automatically estimated negative B-factor resulted with 2.90 Å final resolution. Local resolution changes were calculated with LocRes and rendered with UCSF Chimera. After refinement the particles were divided into five subsets according to the estimated ice thickness value, from each group a set of 7,000 particles was randomly selected and refined again to see how the varying ice affects the final resolution.

#### Gamma-secretase processing

The dataset was processed with Relion3.1 pipeline. Motion correction was done using MotionCor2 with 5x5 patches and binning factor 2. CTFFIND-4.1 was used to estimate the parameters of contrast transfer function. 920,945 particles picked with crYOLO from 2,925 micrographs were used for reference-free 2D classification. The best 2D classes were selected manually. The initial 3D classification resulted with reported resolution 7.47 Å. 308,706 particles from the best 3D classes were used for the 3D refinement with C1 symmetry and resulted in 4.07 Å resolution based on the gold standard FSC = 0.143 criterion. The map was sharpened using a soft mask created from the atomic model PDB 5a63 (Bai et al., 2015) and with automatically estimated negative B-factor. After sharpening, the final resolution was 3.81 Å. The changes in local resolution were calculated using LocalRes. The larger number of particles were kept to allow selection of representative subsets from different estimated ice thickness levels. The particles used for the 3D refinement were were associated with the estimated ice thickness value using the IceBreaker. Three subsets of 60,000 particles each were selected randomly from groups representing thin, medium and thick ice and used for re-refinement and post-processing with the same setup.

### Quantification and statistical analysis

The methods of statistical analysis are provided in method details and supplemental information.

## Data Availability

This paper analyzes existing, publicly available data. These accession numbers for the datasets are listed in the key resources table. The reconstructed cryoEM dennsity maps have been deposited at EMDB and are publicly available as of the date of publication. Accession numbers are listed in the key resources table. The cryoEM density maps reconstructed from T20S proteasome particles picked form various ice-thickness areas have been deposited in the EMDB under accession code EMD-13309 for the group 1 with thickest ice, EMD-13310 for the group 2, EMD-13311 for the group 3, EMD-13312 for the group 4, EMD-13313 for the group 5 with thinnest ice, EMD-13902 for the combined group 4 and 5 and EMD-13901 for the full dataset respectively. The cryoEM density maps from human gamma-secretase particles picked form various ice-thickness areas have been deposited in the EMDB under accession code EMD-13903 for the group 1 with thickest ice, EMD-13904 for the group 2, EMD-13905 for the group 3 with thinnest ice and EMD-13907 for the full dataset. The code has been deposited at Zenodo and is publicly available as of the date of publication. DOIs are listed in the key resources table*.* The software is freely available also from https://github.com/DiamondLightSource/python-icebreaker or can be downloaded with the Python Package Index https://pypi.org/project/icebreaker-em/ Any additional information required to reanalyze the data reported in this paper is available from the lead contact upon request.

## Supplemental information

Document S1. Figure S1

Document S2. Article plus supplemental information

## Footnotes

Supplemental information can be found online at https://doi.org/10.1016/j.str.2022.01.005.
