# Supplementary material for: IceBreaker: Software for high-resolution single-particle cryo-EM with non-uniform ice
Source: Structure. 2022 Apr 7;30(4):522–531.e4. doi: 10.1016/j.str.2022.01.005 (PMC9033277; doi:10.1016/j.str.2022.01.005)
Supplement: Document S1. Figure S1 [file mmc1.pdf]

Structure, Volume *30*

Supplemental Information

IceBreaker: Software for high-resolution single-particle cryo-EM with non-uniform ice

Mateusz Olek, Kevin Cowtan, Donovan Webb, Yuriy Chaban, and Peijun Zhang

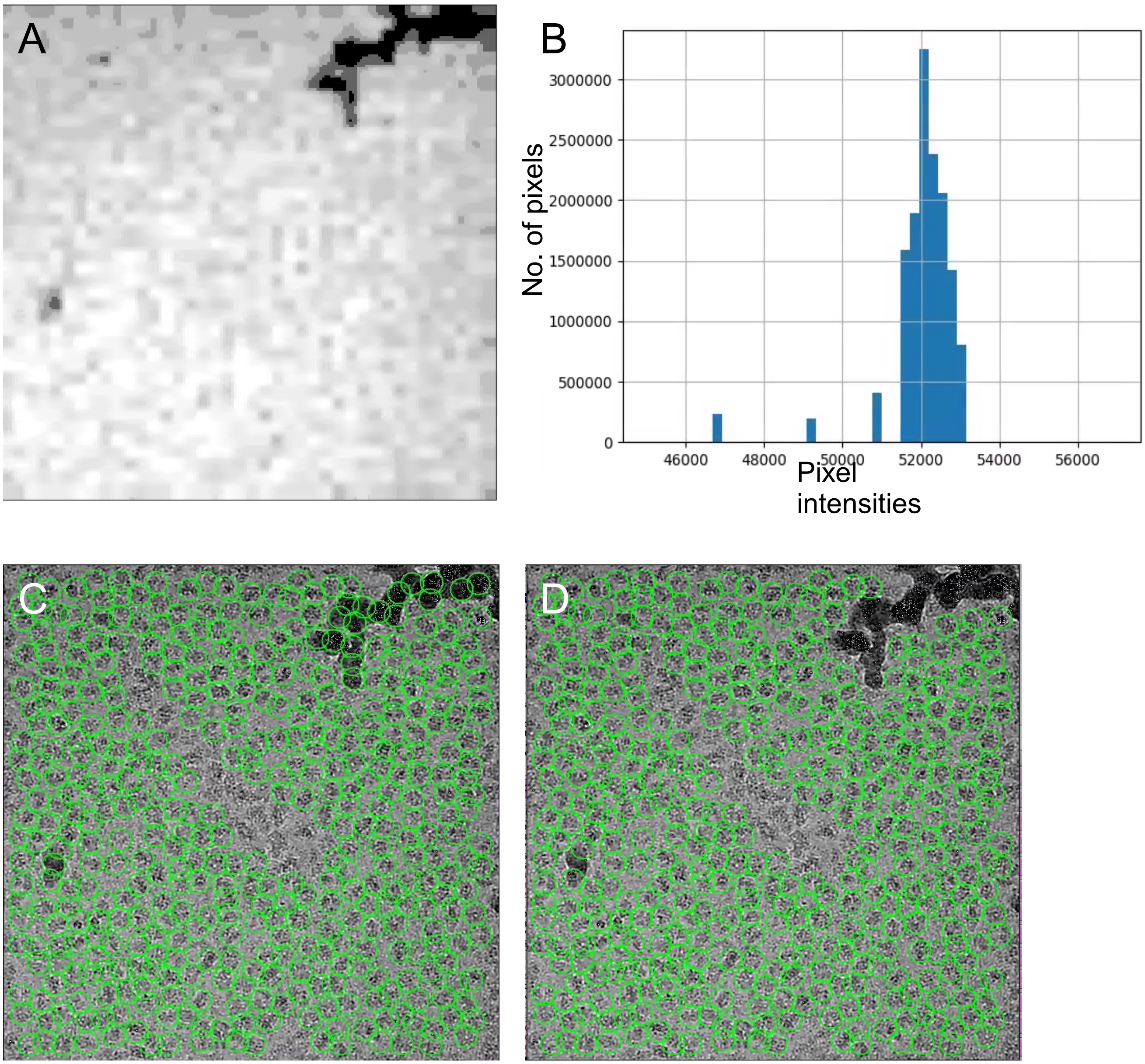

Figure S1 | IceBreaker allows to avoid ice contamination. Related to Figure 3. (A) Segmented micrograph with the ice contamination, (B) histogram of the pixel intensities showing the group of pixels with lower values corresponding to the contamination area, (C) results of the particle picking with template-free Laplacian of Gaussian autopicker, (D) coordinates picked from the ice contamination are removed after applying a threshold based on the pixel intensity distribution.
